# Towards Sustainable Energy-Efficient Communities Based on a Scheduling Algorithm

**DOI:** 10.3390/s19183973

**Published:** 2019-09-14

**Authors:** Carlos Cruz, Esther Palomar, Ignacio Bravo, Alfredo Gardel

**Affiliations:** Department of Electronics, University of Alcala, Alcala de Henares, 28871 Madrid, Spain; esther.palomar@uah.es (E.P.); ignacio.bravo@uah.es (I.B.); alfredo.gardel@uah.es (A.G.)

**Keywords:** cooperative smart community, scheduling algorithm, consumer preferences, renewables

## Abstract

The Internet of Things (IoT) and Demand Response (DR) combined have transformed the way Information and Communication Technologies (ICT) contribute to saving energy and reducing costs, while also giving consumers more control over their energy footprint. Unlike current price and incentive based DR strategies, we propose a DR model that promotes consumers reaching coordinated behaviour towards more sustainable (and green) communities. A cooperative DR system is designed not only to bolster energy efficiency management at both home and district levels, but also to integrate the renewable energy resource information into the community’s energy management. Initially conceived in a centralised way, a data collector called the “aggregator” will handle the operation scheduling requirements given the consumers’ time preferences and the available electricity supply from renewables. Evaluation on the algorithm implementation shows feasible computational cost (CC) in different scenarios of households, communities and consumer behaviour. Number of appliances and timeframe flexibility have the greatest impact on the reallocation cost. A discussion on the communication, security and hardware platforms is included prior to future pilot deployment.

## 1. Introduction

There exists a global aim to conceive novel sustainable services and energy infrastructures to balance supply and demand. Over the last decade, many sustainable development initiatives across the globe have been promoting regulatory campaigns, such as pricing or optional/mandatory thermal retrofit policies, looking at the engagement of cost-effective social behaviour and/or a social pro-environmental morality [1]. To this regard, the Internet of Things (IoT) and Demand Response (DR) combined have transformed the way Information and Communication Technologies (ICT) contribute to saving energy and reducing costs, while also giving consumers more control over their energy footprint [2,3]. Connected devices (e.g, household items, machines, vehicles or gadgets) can automatically influence each other in order to increase the overall potential for energy efficiency and the range of management systems’ involvement.

**Figure 1:**
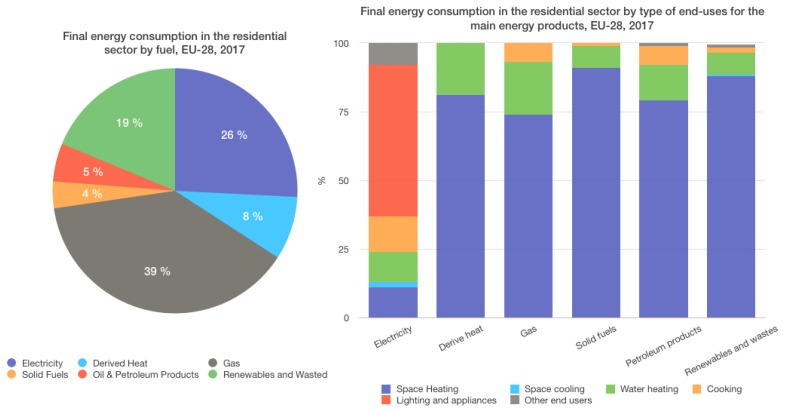
Final energy consumption in the European residential sector from Eurostat survey 2017.

DR programmes, designed to stimulate changes in consumers’ electric usage patterns, thus appear to bolster not only energy efficiency, but also renewable energy resource management initiatives. Current DR strategies are based on providing end-users with individualised tailored advice about their particular habits with incentive payments for load reductions when needed to ensure reliability [4]. For instance, as control and communication technologies become more widely accessible, electricity prices and information are delivered more effectively to consumers. This allows consumers to identify and more easily target discretionary loads that can be curtailed or shifted. On one hand, we can find new challenges to the analysis of these loads and the extraction of consumer/community patterns that produce more automatic and user-friendly DR systems as well as driven by congestion management instead of being price-based. On the other hand, this automation should be enabled by on-site energy controls fed by near-real-time pricing information without significant customer effort or intervention. Furthermore, the real exploitation of renewable sources for energy supply presents multiple challenges not only to utilities, grid and system operators, but also to the consumer that knows very little about its availability or potential from microproduction [5] and energy harvesting processes [6,7]. For instance, according to the Eurostat survey (https://ec.europa.eu/eurostat) (Figure 1), only 19% of the final energy consumption in residential sector comes from renewable resources.

Our proposal intends to bridge the aforementioned gap between utilities and consumers by leveraging consumer cooperation towards a joint daily schedule of their household appliances operation using supply generated from renewable energy sources. In this work, we assume the existence of an Utility entity (a set of energy providers or substations) generating, accumulating, storing and ultimately serving electricity to the consumers. This role, the Utility, is therefore in charge of allocating the available supply from the different energy sources at disposal of the community; it is not, however, dealing with the final destination of the supply (whether to power low-energy electronics or bigger appliances). As an application scenario, imagine a smart community of electricity consumers who, empowered by a better access to their consumption controls and appliance interconnection, are provided with sufficient incentives to coordinate and adjust their energy demands for a certain purpose. These consumption controls are coordinated by a Home Energy Management System, which enables energy management at homes. By doing this, consumers are able to visualise the energy data and make optimum use of energy by controlling their electrical appliances. They autonomously adapt their energy consumption by means of sharing nearly real-time electricity demand information. An aggregator device, capable of shifting the consumers’ use of the resource, will be able to make the overall consumption pursue common goals such as being sustainable, ecofriendly or cheaper. On the other hand, utilities, allowed to perform real-time billing, profiling and fault detection, are also creating incentives for users consuming renewable sources (e.g., guaranteeing the lowest price if the load demand does not exceed a certain threshold). They produce, store, distribute and serve the supply to the consumers who will now benefit from additional information about the supply availability. It has to be observed that neither utilities nor consumers are considering microgenerated energy in the current model. Hence, it is responsibility of the aggregator the computation and rescheduling of the total daily load of the community to avoid overloading the utility supply from renewables. This scheduling represents an optimisation problem whose main factors are the 24 h-vector of the next day’s supply from renewable sources and the duration and activation time preferences of every consumer’s appliance.

In this work, we present a cooperative DR system designed to promote behavioural changes in small or large communities with common interests. The involved entities will reach binding agreements and coordinated behaviour through the aggregator, a device that collects 24-h vectors with the consumers’ demands and the expected supply from renewables. It also centralises the supply allocation algorithm that optimises the distribution of the available green supply between the consumers taking into consideration their time preferences of appliance activation. Experimentation on the algorithm implementation is conducted using estimated values and benchmarks. We include the analysis of the power consumption in watts for most commonly used appliances taking average measures informed by manufacturers; for each appliance type, we show the efficiency label (according to EU normative), the estimated cost while in operation mode and standby mode as well as the average consumption in 24 h time. In addition, we evaluate the algorithm over different strategies of player order selection as well as over the application of four heuristics that optimise the objective search. Evaluation results throw feasible computational cost in all these different programming configurations as well as considering a series of scenarios for household and community settings, and consumer behaviours. Finally, from the empirical results we can discuss on the hardware and networking requirements for an efficient pilot deployment.

The paper is organised as follows. We discuss the related work in Section 2. Section 3 states the system model and design decisions. We describe the simulation of the implemented scheduling algorithm and estimate the performance cost in Section 4. Technical considerations in terms of communication, network protocols, security and hardware platforms are drawn in Section 5. Finally, Section 6 concludes and establishes future research directions.

## 2. Related Work

The starting point of our research can be found in the works by the authors of [8,9], where an adaptive model for DR is envisioned over the deployment of smart meter networks. Special focus is taken on the software design in order to facilitate the integration and scalability of the community system future development. An example of a DR aggregator model is designed in the work by the authors of [10] to facilitate renewable energy integration, where end consumers play a key role.

One of the major challenges in the energy efficiency context is the way to involve end-users in energy markets. This fact can be exemplified in the works by the authors of [11,12], where systems are designed to facilitate DR for residential prosumers. For instance, the work by the authors of [12] shows a system based on an aggregator of residential prosumers that participate in the day-ahead energy market to minimise operation costs by controlling appliances. The performance of an optimisation-based residential energy management scheme is presented in the work by the authors of [13]. This work applies a constrained swarm intelligence model to minimise the total cost of household electricity consumption. As it has been stated by the authors of [14], models based on DR, smart technologies and intelligent controllers can lead to a considerable energy consumption reduction.

The vast majority of the related work addresses energy-efficient solutions and optimisation algorithms from a single consumer/home viewpoint. The appliance scheduling optimisation solution in the work by the authors of [15] considers time ranges and consumer preferences along with different types of appliance consumption profiles. Their solution is based on the Mixed Integer Linear Programming (MILP) technique under “Gurobi” solver, which is addressed to minimise both the total energy cost and the peak load of all the home appliances used per day. This model is unscalable though. Similarly, household load scheduling is also approached in the work by the authors of [16] by the MILP optimisation model. The MILP model and a heuristic algorithm accounting for a typical household user are simulated taking into account overall costs, climatic comfort level and timeliness. MILP is also the technique applied to load shifting by the authors of [17] to optimise the interaction between an aggregator and smart consumers’ operation. The specific DR program incentives and the consumers’ needs are the main parameters that a Smart Home (SH) controller considers to reshape the consumers’ demand profile through shifting the operation of flexible loads. Focused on users’ individual preferences, the work by the authors of [18] sets priorities and preferred time intervals for load scheduling, along with making efforts to optimise the consumption curves of household, commercial and industrial consumers.

A remarkable interaction between the utility and its consumers is modelled through a two-step centralised game in [19], where consumers reduce the peak-to-average power ratio by optimising their energy schedules. The utility supplier pulls consumers in a round-robin (RR) fashion and provides them with energy price parameter and current consumption summary vector. Each user, then, optimises its own schedule and reports it back to the supplier, which, in turn, updates its energy price parameters before pulling the next consumers. Also centralised but considering renewable energy technologies to improve energy efficiency and reduce costs through optimisation algorithms, approaches in the work by the authors of [20] focus on the context of microgrids and storage at residential and commercial building environments. In addition, heuristics based on genetics algorithms [21] and neural networks [22] work on the scheduling of the consumer consumption to save the peak formation. Their simulation results show that the proposed algorithms reduce the peak-to-average ratio and help users minimise their energy expenses without compromising comfort.

Applying a distributed and an autonomous Demand Side Management (DSM) within a neighbourhood, the consumers’ schedulers in the work by the authors of [23] are assumed to be built inside smart meters and connected to the power grid and a local area network. In order to reduce the total energy cost, these schedulers interact automatically by running a distributed algorithm to find the optimal energy consumption schedule. Subscribers also receive incentives to use the schedulers via a novel pricing model derived from a game-theoretic analysis. The authors of [24] formulate a power allocation game, where multiple companies, leaders and their consumers are the followers to reach a unique pure-strategy Nash equilibrium via a distributed algorithm. Authors  find that the multi-period scheme, compared with the single-period one, provide more incentives for energy consumers to participate in DR. For a comprehensive description of the many algorithms that can be used to solve the resource allocation problem, see the work by the authors of [25].

In summary, several optimisation Pareto-efficient approaches to the load and/or consumption adaptive scheduling have been the focus of much attention in demand side management, SHs, wireless sensor networks, broadband networks, and smart grids [26,27].

## 3. System Model

**Figure 2:**
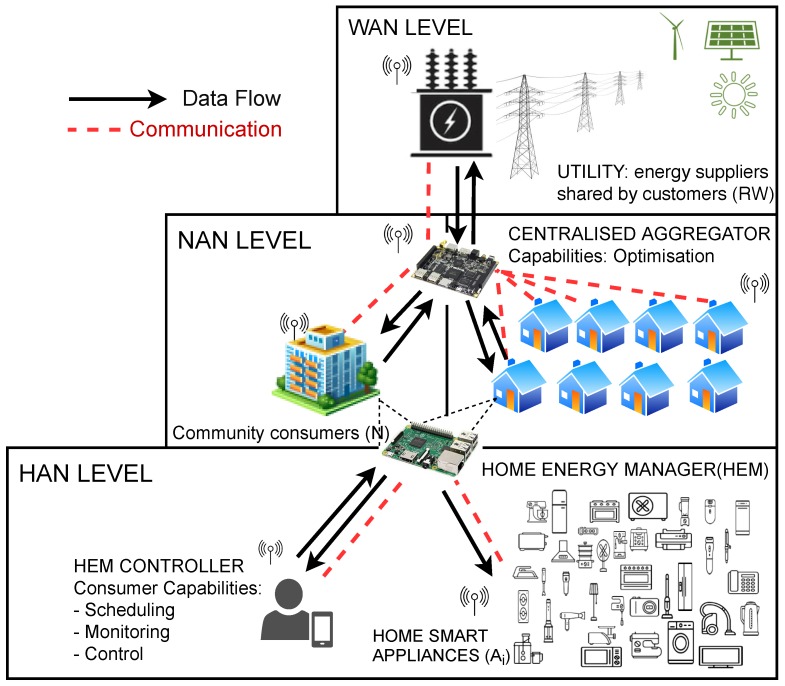
Smart cooperative system divided into Home Area Network (HAN), Neighbour Area Network (NAN) and Wide Area Network (WAN).

Our proposal embraces the use of renewable resources aiming three main actors: *Consumer*, *Aggregator* and *Utility*. Figure 2 illustrates the main roles and processes within the adopted cooperative DR framework.

The first actor, *Consumer*, provides the home energy usage to be managed and automatically controlled by Home Energy Management System (HEMS) that performs three main functions: (1) schedule demand, (2) appliance control and (3) information provider. It selects the daily scheduling preferences, managing a profile for collaboration in a DR system and viewing its account and consume information. The consumer can manage them from a portable device (i.e., an app installed on a mobile phone or tablet) that is connected to a communication network for preferences scheduling. A community will comprise a set of consumers sharing electricity supplier or substation. HEMS pulls scheduling information and generates processed data to the *Aggregator*. HEMS is also responsible for collecting information from the *Aggregator* and controlling a variety of home appliances.

In our proposal, *Consumers* adapt their energy consumption cooperatively on a centralised way, that is, sharing their demand schedule with a data collector, which facilitates the integration of energy consumption information into a common view. This integration is performed over the so-called *Aggregator*, the second actor, which implements an optimised resource allocation algorithm as a response to supply conditions, in particular, targeting renewable sources. The *Aggregator* is defined as the optimal system providing energy management services in order to efficiently manage demand in SH [28]. HEMS acts as a central node and receives the demand scheduled information from the *Aggregator*. Then it loads the power consumption preferences to each appliance and establishes communication for managing the appliances. The *Aggregator* allows the local distribution of the energy provided, according to the availability of renewable resources. This energy management system will be connected to the *Utility*, the third actor, which is a set of energy suppliers shared by customers. We presume utilities implement a distributed generation that allows to gather energy from mainly renewable sources addressed to give lower environmental impacts and improve supply security.

### 3.1. Consumer System Design

Let N denote an ordered set of *Consumers* that are willing to cooperate in the pursuit of global community targets (i.e., becoming greener) by sending their data to the *Aggregator*. Each *consumer*i∈N has a set of household appliances labeled as $A_{i}$. Fixed energy load is identified by factors such as the consumers’ habits, their behaviours and their use of appliances, as well as a variable load resulting from the use of such appliances and other equipments. Formulae and benchmarks can be used to estimate appliance and home electronic energy use in kilowatt hours (kWh) as well as household local records.

Bearing in mind a discrete time slot system, and without loss of generality, we assume that time granularity is one hour per day. Regarding the appliances, each *Consumer* is supposed to preallocate a certain amount of fixed demand and variable consumption planned for the next 24 h [29]. For each appliance, $a_{ij}\in A_{i}$, we assume both daily fixed and variable energy consumption scheduling vectors at each time slot t∈{0,…,23} to control its non-shiftable and shiftable consumption respectively.

We define $fD_{i,a_{ij}}^{t}$ and $vD_{i,a_{ij}}^{t}$ by denoting the corresponding one-hour fixed and variable energy consumptions respectively. Variable energy demand is characterised by its flexibility, as the *Consumer* preference for an appliance starting in a specific period of time is taken into consideration. For each appliance, there is an execution window (i.e., a closed interval) that selects a minimal starting time, and a maximal ending time labelled by $t_{beg}^{i}$ and $t_{end}^{i}$. $t_{sched}^{i}$ is defined as the working time of appliance “i” and matches the range of operation start time $t_{sched}^{i}$ $\in \left[t_{beg}^{i},t_{end}^{i}\right]$. L is defined as the duration of the planned operation of appliance $a_{ij}$ in the next day. Load needs to be switched on for a time between two predefined moments: ∀${}_{ij}$ $\in A_{i}$, $t_{sched}^{i}$≥$t_{beg}^{i}$. In this line, load also needs to be switched off: ∀${}_{ij}$ $\in A_{i}$, $t_{sched}^{i}$≤$t_{end}^{i}$. In other words, *Consumer* *i* will set the following data for its appliance $a_{ij}\in A_{i}$ (see Table 1).

- Fixed consumption (kWh) when appliance $a_{ij}$ is in standby status
- Consumption (kWh) when $a_{ij}$ is on
- Duration (hours/minutes) of the planned operation of appliance $a_{ij}$ in the next day
- Point in time (hour, e.g., 8am) of preferred start of appliance $a_{ij}$ activation
- Point in time (hour, e.g., 12pm) of preferred end of appliance $a_{ij}$ operation

A centralised home controller provides access to all the appliances and devices at home via wireless networks; it will receive and apply the 24-hour reallocated vector from the *Aggregator* to systematically activate/deactivate every appliance without human intervention.

**Figure 3:**
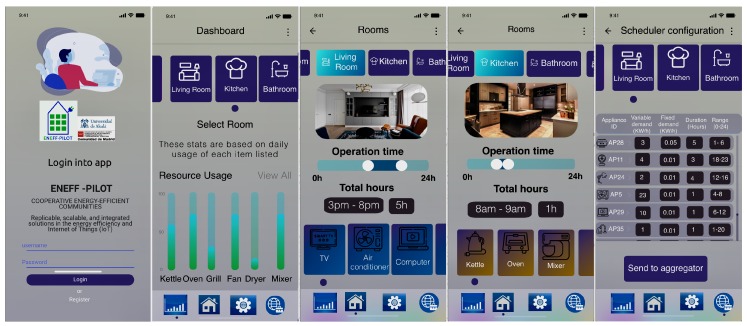
Consumer scheduling app.

Moreover, we have developed an energy consumption scheduling app based on the Adobe XD template [30], which provides the consumer with an interface to control, monitor, visualise and program the functioning of appliances. More specifically, it allows the configuration and setting of the aforementioned data for each appliance $a_{ij}$. Figure 3 depicts a usage sequence to explain how the application works. The app allows users to check the resources used in the previous 24 h as well as to select the appliance in relation to the dwelling zones such as the kitchen or the bathroom, among others. At this stage, consumer will be able to indicate the time range and the duration of activation for each appliance. The last window summarises the introduced demand information. It also provides an estimated power cost in operation and standby for each appliance (according to benchmark analysis in Table 2). Consumers have to give consent by sending these data to the home controller. Finally, a vector is sent to the *Aggregator* with the data structure shown in Table 1.

**Figure 4:**
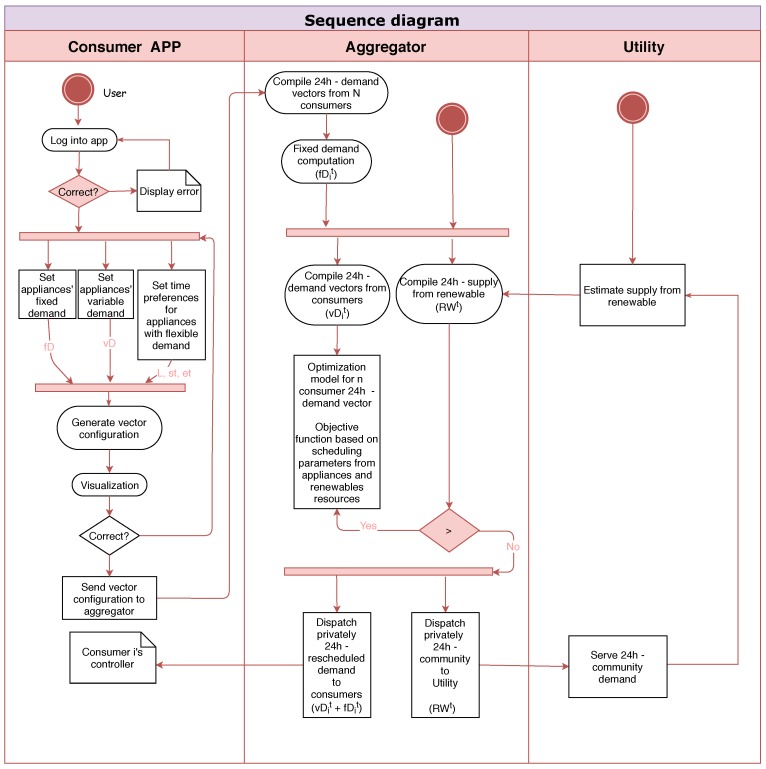
Sequence stating main processes and message exchange among the system’s players.

Data aggregation is defined as a centralised system with aggregation tasks that communicates with the *Utility* and the *Consumers* as shown in Figure 4. An algorithm is originally designed to optimise the allocation of the expected electricity supply from renewables among the community’s *Consumers* related to their chosen preferences.

### 3.2. Aggregator System Design

As an example of a renewable source efficient use, the *Utility* provides essential information on reliable renewable source and fossil energy programmed for the next 24 h by replacing carbon-intensive energy. The energy supply generated from a set of renewable sources at a time slot t∈{0,…,23} is denoted by ${\mathrm{RW}}^{t}$. $fU^{t}$ represents the energy supply at time *t* generated from a set of fossil sources. The *Utility* centralises the distribution of the energy, the notification to the *Aggregator*, and the billing process. The renewable supply vector RW is essential for the *Aggregator* in the optimisation of a fair allocation of such supply between the *Consumers’* fixed (non-shiftable) and variable (shiftable) energy demands.

The daily fixed demand for consumer i∈N is denoted by $fD_{i}=\sum_{t=0}^{23}\sum_{a_{ij}\in A_{i}}fD_{i,a_{ij}}^{t}$ as the aggregated load of non-shiftable local consumption of the appliances and frequent behaviours. The *Aggregator* can then easily compute the daily fixed demand for the whole community of consumers at a time *t* as $fD^{t}=\sum_{i}^{N}fD_{i}^{t}$. On a daily basis, the *Aggregator* verifies that the total energy consumed by all appliances in the system fulfils the daily utility service provided by the *Utility*. It is critical that the community does not reach the worst case such as $\sum_{i}^{N}\sum_{t=0}^{23}fD_{i}^{t}\gg \sum_{t=0}^{23}{\mathrm{RW}}^{t}$. On the contrary, aggregation of the variable energy is more complex given the consumers’ time preferences. The *Aggregator* will execute a fair-share rescheduling of the community’s requested variable demand per hour $vD_{i}^{t}$ aiming at $\forall t\in \left\{0,\ldots ,23\right\},\sum_{i}^{N}\left(fD_{i}^{t}+vD_{i}^{t}\right)\leq {\mathrm{RW}}^{t}$. We will show refinements of the proposed scheduler algorithm looking at the max–min fairness, Pareto-efficiency, envy-freeness, and truthfulness while serving Consumers’ preferences. Perhaps the simplest way to give each Consumer equal chance against all other is to recursively apply a ”round-robin” strategy in the allocation of each Consumer’s needs. Fair random assignment is one of the refinement methods to be compared. A global centralised optimisation problem is faced here, where only a “Nash bargaining solution” is possible such as $\forall i\in \left\{1\ldots N\right\},\mu_{i}^{t}\;=\;fD_{i}^{t}+min\left\{\mathrm{DFC}\left(vD_{i}^{t}\right)\right\}\leq {\mathrm{RW}}^{t}$.

Therefore, solutions to the optimisation problem should satisfy $t_{sched}^{i}$ and L while avoiding overconsumption at ${\mathrm{RW}}^{t}$. The formulation is explained in Algorithm 1 (Demand Calculation Function, DCF) and it will be shaped as its minimum, i.e., $min_{.}\mathrm{DFC}\left(\cdot \right)$ upon request of Algorithm 2. In DFC function, a search for the optimum time slot for every appliance activation takes place given its activation time, its preference interval and the available supply in kW from the renewable utility. In particular, taking into account $A_{i}$, $t_{sched}^{i}$, $t_{beg}^{i}$ and $t_{end}^{i}$ variables, the optimisation will determine how appropriate an adjustment is by minimising the total overconsumption (in hours) of the community appliances against the available renewable supply at a certain time slot.

Finally, upon reaching the optimisation objective, the *Aggregator* will notify the community that an agreement has been reached and privately release the reallocated demand vector ${\vec{\mu }}_{i}\forall i\in N$ to each Consumer.

**Algorithm 1** Demand Calculation Function (DCF)
1:$RW^{t}$(renewable vector) = $\left\{\sigma_{1},\cdots ,\sigma_{24}\right\}$2:
$N\;=\;size(A_{i})$
3:Defining variables $t_{beg}^{i}$, $t_{end}^{i}$4:**for** ihour time to the total number of hours **do**5: **if** ihour doesn’t belongs to interval $\left[t_{beg}^{i},t_{end}^{i}\right]$ **then**6:  $fD_{i}^{t}$ computation7: **end if**8:
**end for**
9:**for** iappliance 1 to size of appliances configuration $\left(A_{i}\right)$ **do**10: ${\mathrm{RW}}^{t}\;=\;{\mathrm{RW}}^{t}--A_{i}(fD_{i}^{t}$)11:
**end for**
12:
$A_{i}\left(vD_{i}^{t}\right)\;=\;A_{i}\left(vD_{i}^{t}\right)--A_{i}\left(fD_{i}^{t}\right)$
13:
$A_{i}\left(D_{i}^{t}\left(D_{i}^{t}<0\right)\right)\;=\;0$
14:$A_{i}\left(fD_{i}^{t}\right)\;=\;A_{i}\left(fD_{i}^{t}\right)--A_{i}\left(fD_{i}^{t}\right)$ Objective Function $F(A_{i},{\mathrm{RW}}^{t},t_{sched}^{i})$**Require:** $A_{i}$ configuration: $vD_{i}^{t},fD_{i}^{t},L_{i},t_{beg}^{i},t_{end}^{i}$**Ensure:** 
$t_{beg}^{i}<t_{end}^{i}$
15:HC initialisation (consume Hourly Energy)16:**for** iappliance 1 to size of appliance configuration **do**17: Set $t_{beg}^{i}$18: Set $t_{end}^{i}$ based on $L_{i}$ and $t_{beg}^{i}$19: **for** ihour time to the total number of hours **do**20:  **if** ihour belongs to interval $\left[t_{beg}^{i},t_{end}^{i}\right]$ **then**21:   $\mathrm{HC}\left(ihour\right)\leftarrow \mathrm{HC}\left(ihour\right)+A_{i}\left(vD_{i}^{t}\right)$
22:  **else**23:   $\mathrm{HC}\left(ihour\right)\leftarrow \mathrm{HC}\left(ihour\right)+A_{i}\left(fD_{i}^{t}\right)$
24:  **end if**25: **end for**26:
**end for**
27:
$\mathrm{RW}{}^{t}s\;=\;{\mathrm{RW}}^{t}--{\mathrm{HC}}^{t}$
28:
$\mathrm{RW}{}^{t}s\left(\mathrm{RW}{}^{t}s<0\right)\;=\;0$
29:
$Demanded\_\mathrm{RW}{}^{t}\leftarrow min\left(\mathrm{RW}{}^{t},\mathrm{HC}{}^{t}\right)$
30:$R1\;=\;sum(\mathrm{RW}{}^{t}s)$; $R2\;=\;max(\mathrm{HC}{}^{t})$31:
Result=sum(R1+R2)
32:
**return** $Result,Demanded\_\mathrm{RW}{}^{t},\mathrm{HC}{}^{t}\left(t_{sched}^{i}\right)$



**Algorithm 2** RR strategy
1:Generate parameters for consumer allocation2:Define global variable RW3:**while** (user < N) and (min(RW) >= 0) **do**4: **if** Optimisation needs **then**5:  Load consumer preferences. $A_{i}$ size from preference array6:  Call Optimisation Function under variables preferences: RW, $A_{i}$, N7:  Number of user ++8:  **if** (RW equals to 0) **then**9:   Break10:  **end if**11: **end if**(No consumer to optimise)12:
**end while**



### 3.3. Proposed Algorithm: A Fair Division Game

The *Aggregator* can apply different approaches to optimisation search within the aggregated load vector vD. It contains all consumer appliances’ scheduling that could be shifted within their preferred activation time frame. This scheduling problem at the *Aggregator* can be seen as a division game given a set of players (either the consumers in N or all its appliances ) and a set of assets (the supply from renewables in RW). In our algorithm we have opted for a turn-based sequential game played by Appliance instead of Consumer for optimisation purposes.

The scheduling problem has to produce a fair division of RW, i.e., a set of rules that, when properly used by the players, guarantees at the end of the game each player will have received a fair share of the assets. In our view, a fair share means that consumers’ preferences on the appliance activation are considered by the *Aggregator* with equity and privacy. As in turn-based sequential games, defining the order under which players start within a turn could be approached in terms of (A) Round-Robin (RR) start: the first player selection policy is RR; (B) random RR; (C) ranking: the first player being the same every time; and (D) randomness: the first player is randomly selected (likewise the sequence order), as follows.

(A) The RR principle, known from other fields such as network scheduling and processor queuing, is based on a process/game/technique, where each task/person/device takes an equal share of something in turn. The RR scheduling can allocate the available electricity from renewables both simple and fairly among the *Consumers/ Appliances*, because (1) the consumers’ number is known and fixed and (2) the reallocation process is centralised by the *Aggregator* which, starting on its own, will satisfy the demand of the *Consumers/Appliances* in a periodically repeated order. We include pseudocode of our algorithm’s main function in the round robin strategy, being the rest pseudocodes similar with exception of the player turn selection on Algorithm 2-line 3. RR results in max–min fairness if the *Consumers/Appliances’* demands are equally sized; otherwise, fair queuing that establishes a fair share size would be desirable.
(B) A random RR scheduling: A similar process as in A), though the election of first *Consumer* is random.
(C) A picking-sequence has several merits as a fair division protocol [31]. Assuming that each agent has a (private) ranking over the set of objects, the allocator must find a policy (i.e., a sequence of agents that maximises the expected value of some social welfare function). Moreover, picking sequences are a natural way of allocating (indivisible) items to agents in a decentralised manner: at each stage, a designated agent chooses an item among those that remain available. The goal of the method is to identify the fairest sequence.
(D) A random process could, or could not, introduce efficiency (no other “random” assignment dominates) in the aforementioned methods while keeping them Pareto-efficient, envy-free and giving good approximation to the social welfare. Efficiency in terms of computational time is also at stake.

## 4. System Validation

In this section, we measure the Computational Cost (CC) of the implemented scheduling algorithm evaluating the suitability of a number of four heuristics applied to the optimisation search and on a series of different case scenarios of consumer communities. In particular, the evaluation of the heuristics and their behaviour on our algorithm under the same input parameters will assist in the selection of the hardware platform for an efficient deployment.

### 4.1. Optimisation Algorithms Used

We adopt heuristic techniques to perform a partial random search of optimal solutions to our objective, i.e., either when the reallocation demand is met or when the number of predefined iterations is reached. We have identified and implemented the following four optimisation methods to guide the search of a workable solution, i.e., the nearest local minimum standard strategy in our Algorithm 1 as follows.

(i) Simulated Annealing (SA) [32] finds a local minimum solution for our Algorithm 1 (DCF) starting at an initial operation time $t_{sched}^{i}$. As explained in Algorithm 3, SA starts generating trial point based on current estimates and evaluates the function by accepting a new value generated after T parameter is set. The solution must consider the $\left[t_{beg}^{i},t_{end}^{i}\right]$ time constraints. $t_{sched}^{i}$ can randomly generate and filter by L. In case of better D, the original one $D^{\prime }$, $t_{sched^{\prime }}^{i}$ could be accepted as better solution if $D^{\prime }$ is worst than D. After the internal counter reaches its threshold, T is cooled down and re-select the best solution again with the reset counter.
**Algorithm 3** Optimisation based on SA algorithm1:Let T> 0 as initial parameter2:Let N(T) as maximum number of iterations3:**while** stop criterion has not been met **do**4: Randomly generate a fasible solution $t_{sched}$5: Evaluate $t_{sched}^{i}$, D = f($t_{sched}^{i}$)6: n = 17: **while** while n <= N(T) **do**8:  Generate solution $t_{sched^{\prime }}^{i}$ based on $t_{sched}^{i}$9:  Evaluation of $t_{sched^{\prime }}^{i}$; $D^{\prime }$ = f($t_{sched^{\prime }}^{i}$); δ =  f($t_{sched^{\prime }}^{i}$)–f($t_{sched}^{i}$)10:  **if** f($t_{osi}^{\prime }$) < f($t_{sched}^{i}$) **then**11:   $t_{sched}^{i}$ = $t_{sched^{\prime }}^{i}$12:  **else**13:   **if**δ >= 0 and u< exp((f($t_{sched^{\prime }}^{i}$)–f($t_{sched}^{i}$))/T) **then**14:    $t_{sched}^{i}$ = $t_{sched^{\prime }}^{i}$15:   **end if**
16:  **end if**
17:  n = n+118: **end while**
19: T reduction and update $t_{sched}^{i}$ at each reduction20:**end while**
(ii) Genetic algorithm (GA) [33] is identified as a method mainly used to solve optimisation problems based on a natural selection process similar to biological evolution. GA finds an optimal operative time from our Algorithm 1 (DCF) for the $A_{i}$ variables. As explained in Algorithm 4, GA can find a solution beginning with random population of points. GA repeatedly modifies a population of individual solutions. At each step, GA produces a next generation population based on a randomly selection of individuals from the current population. After that, the population turns into an optimal solution. The evaluation number is increased when the method finishes by calculating one generation P. Each generation is a feasible solution for the appliance scheduling ($t_{sched}^{i}$ per appliance). In the evaluation stage, the best solution $t_{sched}^{i}$, which has the lowest demand, is inserted to the best solution set. Mutation and crossover operators are selected to generate the next evaluation from the current generation. The mutation operator randomly shifts the scheduled start times of some appliances in order to generate newly solutions that may have a better result in demand efficiency. They are screened with the constraints to filter out the infeasible ones. The crossover driver swaps scheduled $t_{sched}^{i}$ under feasible solutions.
**Algorithm 4** Optimisation based on GA algorithm1:Generate Solutions. Build a set of PopSize P solution2:Reformulation of solutions. Selection of a local search method to each solution in P3:**while** number of evaluations < MaxEval **do**4: $t_{sched}^{i}$ introduction to P. Evaluation of solution in P and update5: Probability of survival based on the quality of the solution6: P solution is partially selected to apply the mutation and crossover operation7: Number of evaluation ++8: Constraint validate P for each $t_{sched}^{i}$. Discard solutions which are disqualified9:**end while**
(iii) Pattern Search (PS) [34] polls the values around the current point and determines the direction that will minimise our Algorithm 1 (DCF) starting at an initial operation time $t_{sched}^{i}$. For each possible direction, an all linear combination of the current position is created, and each pattern is multiplied by the size of the mesh to obtain a new one. As presented in Algorithm 5, PS investigates nearest neighbourhood of a possible solution always in the range of lower and upper bounds $\left[t_{beg}^{i},t_{end}^{i}\right]$ for each appliance. This solution seeks to find a better one. A failure improvement generation by neighbours (L and D) would reduce the search step (Δ). Search finishes when the step gets sufficiently short, ensuring the convergence to a local minimal overconsumption.
**Algorithm 5** Optimisation based on PS algorithm1:Initialise predefine default search step $\Delta_{0}$; $t_{sched}^{i}$ and Δ = $\Delta_{0}$2:**while** Termination condition not reached **do**3: init current solution D= ($t_{sched}^{i}$+L*Δ)4: Evaluate nearest neighbours in D5: **if** betters in D **then**6:  Update the current solution to the best neighbour in D; Δ = $\Delta_{0}$7: **else**
8:  Search step reduction Δ = $\Delta_{0}/2$9: **end if**
10:**end while**
(iv) Particle Swarm Optimisation (PSO) [35] is a stochastic search method and simulates the social behaviour of particles used to find parameters that minimise a given objective. The optimisation determines the minimum value and the best location evaluating our Algorithm 1 (DCF) through iterations.

Algorithm 3 illustrates this search procedure, which is initialised with the generation of particles assigning initial velocities and positions. The operative appliances time is defined as a set of lower and upper bounds $\left[\vec{t_{beg}},\vec{t_{end}}\right]$, where the solution is found in operation time range $\vec{t_{beg}}$ = $\left(t_{beg}^{i1},...,t_{beg}^{ij}\right)$, and $\vec{t_{et}}$ = $\left(t_{end}^{i1},...,t_{end}^{ij}\right)$. The vectors $\vec{x}$ = $\left(x^{i1},...,x^{ij}\right)$ and $\vec{v}$ = $\left(v^{i1},...,v^{ij}\right)$ are the current position and velocity, respectively. Each individual adjusts its position according to a linear combination of its inertia ω, the best location of individual particle $\vec{p}$ = $(p^{i1},...,p^{ij}$) and the best location of particle swarm $\vec{g}$ = $(g^{i1},...,g^{ij}$). The confidence degree is determined by the random operators $\phi_{p}$ and $\phi_{g}$ in the range [0,1] together with the confidence coefficients $c_{p}$ and $c_{g}$. They are responsible for moving in the direction of the best position of a particle and the global best position. The new displacements are no more than one way of trying to imitate other individuals. It then iteratively updates the solution positions (the new location is the old one plus the velocity, modified to keep particles within $[\vec{t_{beg}},\vec{t_{end}}]$, velocities and neighbours). The solution, above $A_{i}$, tries to find the optimal ones. After several iterations, particles converge to the best solution.

**Algorithm 6** Optimisation based on PSO algorithm
1:Initialise population of particles with random values positions in the search space $\vec{x}$∼U$\left[\vec{t_{beg}},\vec{t_{end}}\right]$2:Set each particle best known position to its initial position $\vec{p}$←$\vec{x}$3:Initialise each particle velocity to random values $\vec{v}$∼U[$\vec{-d}$,$\vec{d}$] where $\vec{d}$ = $\vec{beg}--\vec{end}$4:Initialise the best known position $\vec{g}$ to the $\vec{x}$ where $f(\vec{x}$) is lowest5:**while** Termination condition not reached **do**6: **for** Each particle *i* **do**7:  **if** i>1 **then**8:   Choose two random numbers $\phi_{p}$,$\phi_{g}$9:   Adapt velocity $\vec{v}$←ω $\vec{v}$ + $c_{p}$ $\phi_{p}$($\vec{p}$–$\vec{x}$) + $c_{g}$ $\phi_{g}$($\vec{g}$–$\vec{x}$)10:   Bound $\vec{v}$ for all dimensions i ($\vec{v}$, -$\vec{d}$, $\vec{d}$)11:   Update the position of the particle $\vec{x}$←$\vec{x}$ + $\vec{v}$12:   Bound population $x_{i}$ for all dimensions i ($\vec{x}$,$\vec{t_{beg}},\vec{t_{end}}$)13:  **end if**14:  **if** $f\left(\vec{x}\right)<f\left(\vec{p}\right)$ **then**15:   update the particle’s best known position $\vec{p}\leftarrow \vec{x}$16:  **end if**17:  **if** $f\left(\vec{x}\right)<f\left(\vec{g}\right)$ **then**18:   update the particle’s best known position $\vec{g}\leftarrow \vec{x}$19:  **end if**20: **end for**21: $\vec{g}$ holds the best found position in search space22:
**end while**



### 4.2. Performance Analysis

Simulation runs on a computer with the following specifications: CPU: 2.3 GHz Intel Core i5; Memory: 8 GB 2133 MHz LPDDR3 and MATLAB R2018b [36]. We evaluate the computational cost of the proposed Algorithm 1 on a series of experiments that represent a variety of possible scenarios of community sizes, consumption patterns or consumer behaviour as depicted in Table 3. Experimentation will help us to identify the most influential factor/s in the computation of the community scheduling.

**Figure 5:**
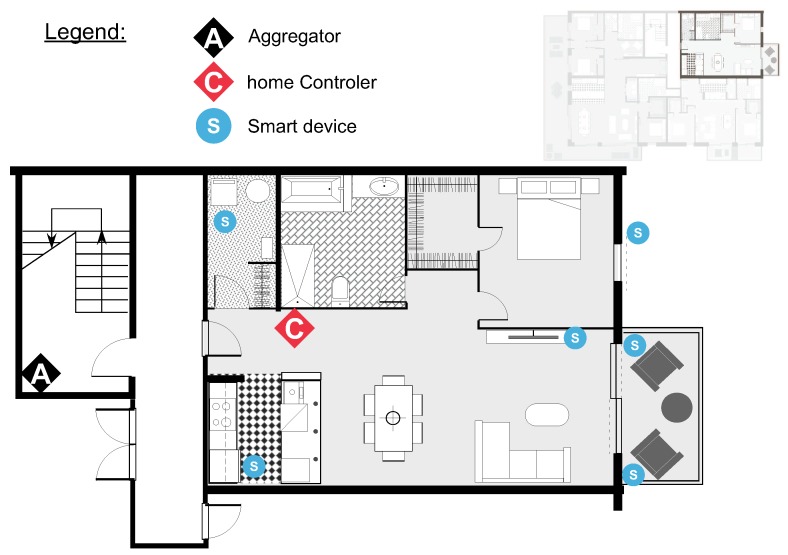
Distribution of the appliances, consumers and aggregator in the community.

We have conducted an analysis on the most common appliances’ real consumption estimation from manufacturers and data sources from the authors of [37], the U.S. Department of Energy (http://www.energy.gov/), the National Grid report (http://www.nationalgrid.com), the authors of [38] and the reports (https://standby.lbl.gov/docs) as well as the manufacturer data to set the scenarios. Our benchmark is depicted in Table 2. Scenarios were envisioned from the design of a residential building as in Figure 5. In particular, we have generated eight scenarios as illustrated in Table 4 and conducted hundreds of experiments for the different factor values to obtain results on a boxplot shape. On the one hand, we can denote as altruistic or flexible a consumer whose time preferences range is big (e.g., from 0h to 23 h); such types of communities are represented by Cases 1, 3, 5 and 7. On the other hand, Cases 2, 4, 6 and 8 illustrate communities on a more selfish setting. Duration is set equally in both situations.

We will compare our method’s performance with the four different heuristics mentioned in Section 4.1, i.e., SA, PSO, GA and PS, and evaluate the efficiency of the different strategies presented in Section 3.3 on the search for the optimisation objective.

**Figure 6:**
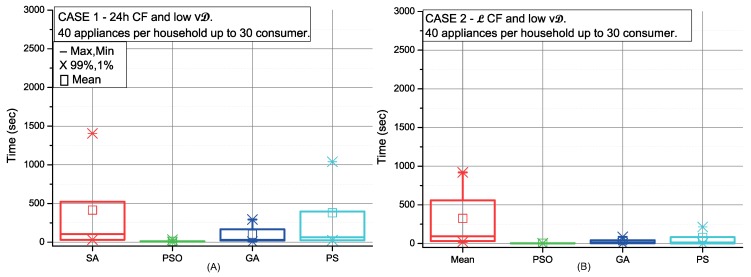
Comparison of the SA, PSO, GA and PS methods for low variable and high fixed consumption in 24 h CF (**A**) and L CF (**B**).

**Figure 7:**
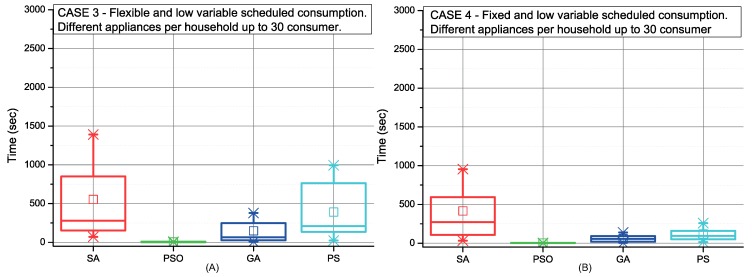
Comparison of the SA, PSO, GA and PS methods for low variable and high fixed consumption in 24 h CF (**A**) and L CF (**B**).

**Figure 8:**
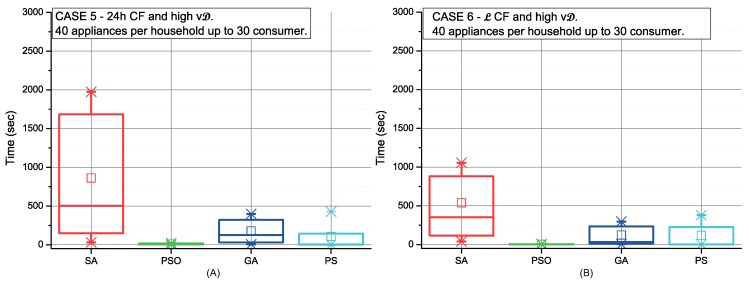
Comparison of the SA, PSO, GA and PS methods for both high variable and fixed consumption in 24 h CF (**A**) and L CF (**B**).

**Figure 9:**
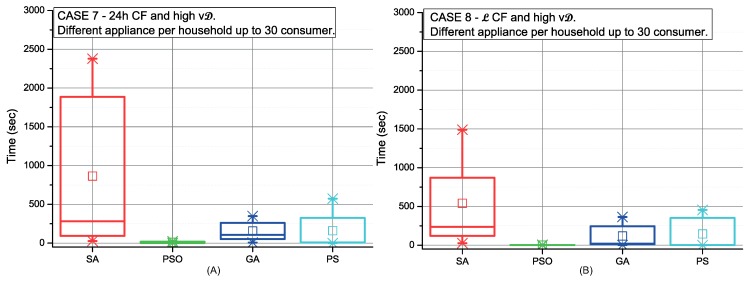
Comparison of the SA, PSO, GA and PS methods for both high variable and fixed consumption in 24 h CF (**A**) and L CF (**B**).

Figure 6, Figure 7, Figure 8 and Figure 9 depict the scheduling cost for the different case scenarios of consumers using strategy C picking-sequence. These scenarios represent extreme conditions either considering high number of appliances and/or an uneven distribution of them, and also the flexibility of the consumers’ time preferences. Communities with selfish settings or fixed consumption display the best results over all different optimisation procedures (cases 2, 4, 6 and 8).

The CC is higher when consumers have an uneven number of appliances. This effect can be observed in Figure 7 and in comparison with Figure 6. The same occurs in scenarios with high variable demand (see Figure 9 and Figure 8). In addition, a high variable demand (Figure 8 and Figure 9) penalises the CC when compared with low settings (Figure 6 and Figure 7). We find the worst case situation on altruistic communities with high variable demand when applying strategy C under SA optimisation. As Figure 9 (red colour) shows, it takes 30 minutes. Both PSO and GA work with sets of solutions that interact between themselves. Both perform better due to the number of solutions managed at the same time. We can also conclude that strategy C under all possible scenarios can be solved within the next 24 h, being PSO the most computationally efficient for scheduling (28sec).

**Figure 10:**
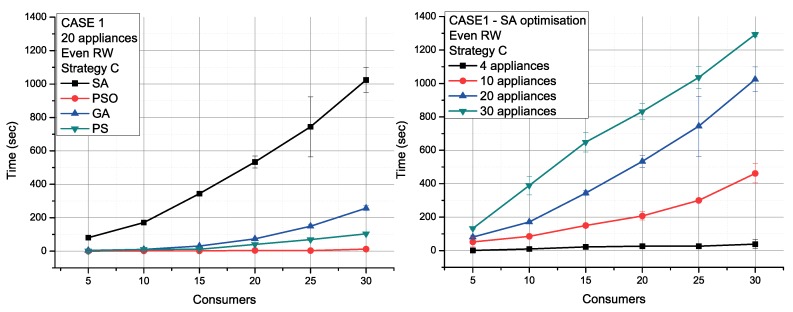
Different approaches (**left**) and appliances number results (**right**).

Additional simulation measures the performance of communities of 20 appliances per consumer in Case 1. Figure 10 (left) compares all the algorithms and shows that PSO achieves a global optimum solution quickly. GA obtains similar outcomes. Applying SA, Figure 10 (right) shows that the cost needed for the scheduling increases linearly with the number of appliances.

**Figure 11:**
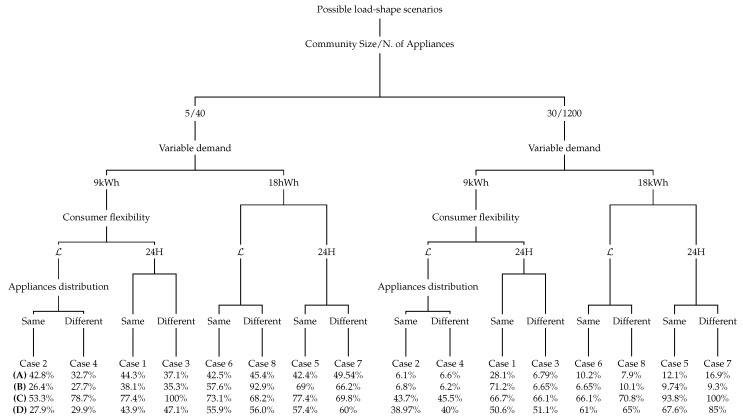
CC results (in %) after applying different strategies (Section 3.3) performed under SA: Round-Robin (**A**), Randomly Round-Robin (**B**), “having the first consumer always the same” (**C**) and randomly (**D**).

So far, experiments have been mainly focused on strategy C. Figure 11 depicts the optimised cost obtained after applying all strategies, and taking into account the different factors (see Table 4). These factors are differentiated by branches and data are expressed as a percentage of the required CC.

Strategy C performs badly, with higher CC in all circumstances. This is mainly because the *Aggregator* needs more resources when it has to optimise all consumers and their appliances all together. The simulation performed with consumers by adding their preferences randomly (strategy D) shows similar cost when compared to strategy C. By contrast, the *Aggregator* under strategy A optimises consumers preferences consecutively in an individual way. A new variant of RR is to perform this strategy when the first consumer starts randomly (strategy B). Both strategies act equally, though dispersed. Strategy A appears as the most appropriate strategy on our system.

Further conclusions can be extracted globally for all the strategies. The CC is higher in four possible situations: when users set a very flexible demand need, when the community is large, when the number of appliances is also large and when a high demand is needed (Figure 11, last branches). A better performance is achieved under strategy B when consumers demand low variable load, in a selfish and small community (Figure 11, first branch). The distribution of appliances also impacts the CC, being higher in large communities with uneven number of appliances per neighbour. The highest CC, which exceeds the half an hour of computation, is obtained in strategy C scenario under a high demand flexibility for an optimisation of 30 neighbours with different appliances distribution per dwelling (Figure 11, last branch).

**Figure 12:**
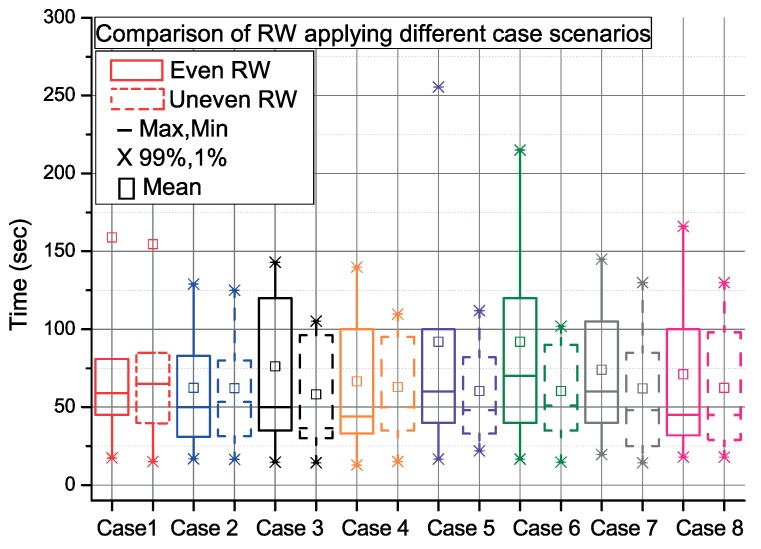
Comparison of RW factor from cases 1 to 8 by applying strategy A under SA.

Finally, Figure 12 compares the CC considering two different RW vector structure provided from the available sources at the *Utility*: uniform RW vector and the 50% standard deviation of RW values. For the eight different cases, and using strategy B and SA, testing is performed for communities of 5–30 consumers. In terms of the chosen strategy, both situations display similar behaviours.

## 5. Technical Considerations: Communication, Security and Hardware

Discussion on the development of a pilot testbed for our system over the existent smart home technologies, their security properties and more feasible communication protocols are included in this section. Extensive work on networking infrastructures has been proposed for smart metering data transmission [14,39]. Some approaches focus on fiber-optics for a high-speed data exchange transmission [40], whose deployment cost would only worth when high data transmission rates are required. Power Line Carrier (PLC) is generally applied to computer networks, wired smart meters among other purposes such as remote monitoring and direct control applications offered by utility companies to consumers [41,42]. Note that regulation should be taken into account to allow the use PLC technologies in outdoor deployments as discussed in the work by the authors of [43].

Infrastructures in a Home Area Network (HAN) comprise the communication technologies for deploying HEMS integrating the household appliances. Communication protocols for data transmission between appliances can be provided with a variety of unwired techniques [28] such as (1) *ZigBee*, which offers an adequate range communication with low data rate and power consumption; (2) *Z-Wave*, which has been used for short range communication due to the low latency communication of small data packets in scalable environments; (3) *6LoWPAN*, which can be applied to building automation designs [44] and to home automation architectures [45]; (4) *Bluetooth*, which is widely used to exchange data over short distances in low energy usage and fast data exchange [46]; and (5) *GSM* networks and *WLAN*, which provide low latency robust communications [47].

Neighbourhood Area Network (NAN) connects customers’ HEMS on a two-way communication infrastructure responsible for transmitting their demands and time preferences to the *Aggregator*, as well as the traditional control messages and power grid sensing data. Wireless cellular is widely used in this type of scenarios as described in the work by the authors of [14].

A Wide Area Network (WAN) establishes communication between the *Aggregator* and the Utility substations. Distance to cover is in a radius of a thousand meters comprising of two interconnected networks [48]. Protocols *LoRaWan* and *5G* demonstrate high speed, bandwidth and responsiveness while operating on various licensed and unlicensed frequency bands. Moreover, *LPWAN (LoRa)* will fulfil most of the IoT challenges and applications. By contrast, the introduction of *5G* into the IoT world is still slow and other technologies sound more promising at present time. Table 5 summarises the main features of the discussed technologies and includes recommendations on more appropriate application areas.

In terms of security and privacy, HEMS involve the deployment of physical controls, cyber-security countermeasures as well as privacy leakage prevention [49]. In addition, a gateway architecture for high system availability is proposed in the work by the authors of [50]. Anonymous authentication applying zero-knowledge proof of knowledge could be the solution to provide anonymous authentication between consumers and *Aggregator*. The latter needs to guarantee compliance with the General Data Protection Regulation (GDPR). Furthermore, a methodology to assess the security risks within the HAN domain should be developed as in the work by the authors of [51]. Further details can be found in the work by the authors of [52], where the authors explain the different IoT security threats scenarios (e.g., personal information leak) and provide an evaluation method within a situational smart home framework.

Table 6 identifies the most promising hardware platforms to build our HEMS emphasising low-cost, compatibility, scalability, easy programming and lightweight properties [48,53]. Raspberry Pi 3 [54] is a single-board computer with integrated Bluetooth and WiFi module and enough resources to control the smart appliances and send/receive our system messages. The emergence of cheap microcontrollers like the Arduino has enabled the implementation of low-cost HEMSs mainly devoted to obtain the consumption data as to generate demand/load profiles [55]. For example, the work by the authors of [56] designs and implements a remotely controlled, energy-efficient and highly scalable HEMS using Zigbee in Arduino Mega board as a central controller. In [57] it is discussed and evaluated the performance of *BeagleBone blue* for HEMS developments, an open-source hardware platform with similar principles of Raspberry Pi. Similarly, the proposal by the authors of [58] develops a remote monitoring system using “Libelium Waspmot”, a modular device that allows the integration of different sensors and radio transceivers. Additionally, deep learning implementations on Field Programmable Gate Array (FPGA) performs fast due to the exploration of parallel computing [59]. Particularly in the work by the authors of [60], Zedboard implementation (Zynq-ARM Cortex-A9 processor) allows the control of unpredictable loads in a deterministic demand management model. In the work by the authors of [61], the algorithm is modelled in Verilog language on a FPGA allowing dynamic reconfiguration of the HAN. A HEMS prototype is developed on a Cubietruck board (Linux based cortex A7 processor) using a WiFi module [62]. It transmits real-time sensing data using TCP/IP communication protocols.

In terms of our DR system, and from preliminary design decisions, our prototype will consist of a Raspberry testbed as the main processor of the HEMS, as it offers good computing performance at a very low price. Its interoperability will provide the performance of alternatives protocols such as ZigBee, WiFi or Z-wave. In the proposed architecture, the WiFi wireless communication between the Aggregator, HEMS and appliances can transfer the data at around a hundredth millisecond, a suitable speed for our current application. The proposed centralised DR system (*Aggregator*) will also operate in an open-source HW platform.

## 6. Conclusions

Globally, smart communities are envisioned more efficient as residents gain autonomy and self-organisation for reducing and shifting any resource consumption. Strategies for energy demand response applied to smart residential communities can lead to improved scenarios of energy efficiency. Consumers have the opportunity to reduce their electricity cost and/or peak-to-average ratio through scheduling their power consumption. In this article, we have described a DR model that integrates the electricity supply available from renewable energy sources into the scheduling process, which is centralised via the community *Aggregator*. We have showed details of community scheduling algorithm implementation and evaluated it in terms of its computational cost. Empirical comparison of our algorithm design on different implementation strategies for player turn selection and optimisation heuristics as well as on a series of case scenarios of community’s consumption patterns showed feasible results in all cases (less than 1 minute to compute the rescheduled community vector). Simulations are conducted with data from our own benchmark of appliance power cost. We have also illustrated development decisions of a mobile app for consumers introducing their demands and time preferences. Finally, we included the discussion of the preliminary decisions on the hardware requirements and communication protocols for a pilot deployment. Immediate future work includes the pilot deployment comprising the algorithm/Aggregator running on the most suitable HW platform as well as the home controllers that autonomously activate/deactivate the smart appliances. We also plan to refine the scheduling algorithm as to consider the usage or purpose of the consumption along with the device type. Furthermore, Utilities and Aggregators in possession of the real-time data from microgeneration and other energy harvesting generators would enhance the conceptual demand response model. Finally, a study of the community patterns will be conducted through game theory and evolutionary computation methods.

## Figures and Tables

**Table 1:** Appliance configuration.

Appliance Configuration				
Consumption (kWh)	Fixed consumption (kWh)	Duration (hours)	Time ON	Time OFF
$vD_{i}^{t}$	$fD_{i}^{t}$	L	$t_{beg}$	$t_{end}$

**Table 2:** Common household appliance energy use.

ID	Appliance	Model	Watts (W)	Efficiency Ranges European Union $A,A^{+},A^{++},A^{+++}$	Estimated Average Power in 24 h (kWh)	Estimated Standby Power in 24 h (kWh)	Estimated Operative Time in 24 h (h)
AP1	Water Heater	Wesen ECO30	2000	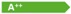	10–14.73	0.010	1–15
AP2	Clothes Dryer	Balay 3SB285B	4350	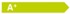	1–2.22	0.015	1–10
AP3	Clothes Washer	Eutrotech 1106	1800	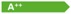	1–2.67	0.015	0.5–10
AP4	Iron	Rowenta DX1411	2100	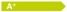	0.1–3	0.002	1–3
AP5	Air conditioner	Fujitsu STG34KMTA	9400	-	3.9–24.3	0.015	0.3–15
AP6	Room air conditioner	Rinnai RPC26WA	2600	-	8–24.3	0.015	3–18
AP7	Heater	DeLonghi HSX3324FTS	2400		1–7	0.08	0.1–10
AP8	Fan heater	Dyson AM09	2000	-	1–6.7	0.015	0.1–10
AP9	Dehumidifier	DeLonghi DEX	210	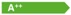	4–24.3	0.005	1.1–9
AP10	Electric blanket	Medisana HDW	120	-	1–3	0.08	1.2–9
AP11	Ceiling Fans	Westinghouse Bendan	80	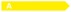	0.5–9	0.01	0.5–5
AP12	Attic Fans	Remigton	500	-	4.73–6	0.01	0.1–18
AP13	Tower Fan	Sunbeam FA7250	40	-	1.4–3	0.03	0.1–18
AP14	Hoover	BGLS4TURBO	750	-	3–6	0.02	0.3–18
AP15	Boiler	Greenstar Ri	9000	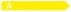	8–22	0.05	0.1–3
AP16	Coffee maker	DeLonghi ECOV	1100	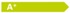	9–12	0.05	0.1–3
AP17	Refrigerator	Bosch KDN46VI20	500	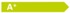	8.77–10	0.05	4.77–24
AP18	Dishwasher	Bosch SMS88TI36E	1500	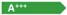	0.5–1.5	0.015	0.3–4
AP19	Food processor	Becken BFP-400	110	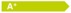	0.5–2	0.015	0.1–5
AP20	Freezer	Bosch GSN36BI3P	350	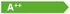	6–8	0.009	0.1–24
AP21	Microwave	Balay 3CG5172N0	1700	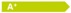	0.9–3	0.01	0.1–4
AP22	Oven	Bosch VBD5780S0	5000	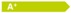	10.96–12	0.01	0.1–8
AP23	Toaster	Russell Hobbs 21973	1100	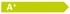	0.2–1	0.01	0.1–1
AP32	Lighting	Osram	100	-	0.7–3	0.01	0.1–24
AP25	Vaporizer	Philips GC362/80	400	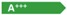	0.3–2	0.07	0.1–8
AP26	Printer	HP Officejet 3833	100	-	0.8–1	0.05	0.1–4
AP27	Computer	Samsung ls24a450	350	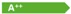	0.7–15.3	0.05	0.1–24
AP26	TV	Panasonic TX43E302B	54	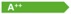	0.1–100	0.05	0.1–24
AP29	Kettle	Philips HD4644/00	3000	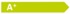	6–19	0.01	0.1–1
AP30	Security Alarm	Vbestlife	20	-	0.6-1	0.02	0.1-24
AP31	Auto Cook	MUC88B68ES	1200	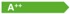	1–3	0.09	0.1–3
AP32	Air Cleaner	Balay 3BC598GN	150	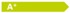	1.1–6	0.01	0.1–6
AP33	Vacuum Cleaner	Hoover TH31HO01	1000	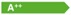	0.9–3	0.06	0.2–4
AP34	Electric Fryer	DeLonghi F26237	1800	-	13–16	0.05	0.2–3
AP35	LedTV	LG 49LJ515V	250	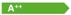	1.9–5	0.05	0.2–24
AP36	Electric Store	Dura Heat EUH4000	4000	-	2.4–4	0.05	0.3–23
AP37	Speaker	Logitech Z120	180	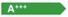	0.3–4	0.01	0.2–20
AP38	Hair Dryer	Rowenta CV3812F0	2100	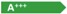	0.99–4	0.01	0.2–6
AP39	Smart Camera	Yi Home	4	-	0.99–2	0.01	0.2–24
AP40	Monitor Sensor	iHome	5	-	0.99–10	0.01	0.1–24

**Table 3:** List of factors for the different case scenarios.

Factor	Type	Value
Community Size	High, Low	30, 5
N. of Appliances	High, Low	1200, 40
Distribution of Appliances	Same, Different	S, D
Fixed Demand	High, Low	Not influenced by optimisation
Variable Demand	High, Low	Up to 18 kWh${}^{6}$, Up to 9 kWh${}^{6}$
Consumer Flexibility	High, Low	24 h, A duration: L
Vector of RW	Even, Uneven	10 kWh, [10 kWh–20 kWh] 50% SD

**Table 4:** Possible load-shape situations.

	Community Size N	N. of Appliances A	Distribution of Appliances	Fixed Demand fD (kWh)	Variable Demand vD (kWh)	Consumer Flexibility CF	RW Vector per Hour (kWh)
Case 1	From 5 to 30	From 40 to 1200	S	Up to 0.43	Up to 9	24 h	10
Case 2	From 5 to 30	From 40 to 1200	S	Up to 0.43	Up to 9	L	10
Case 3	From 5 to 30	From 40 to 1200	D	Up to 0.43	Up to 9	24 h	10
Case 4	From 5 to 30	From 40 to 1200	D	Up to 0.43	Up to 9	L	10
Case 5	From 5 to 30	From 40 to 1200	S	Up to 0.43	Up to 18	24 h	10
Case 6	From 5 to 30	From 40 to 1200	S	Up to 0.43	Up to 18	L	10
Case 7	From 5 to 30	From 40 to 1200	D	Up to 0.43	Up to 18	24 h	10
Case 8	From 5 to 30	From 40 to 1200	D	Up to 0.43	Up to 18	L	10

**Table 5:** Wireless networks.

Technology	Standard	Data Rate	Frequency Band	Power Consumption	Complexity Transmission Range	Strengths	Application Areas	Encryption/Authentication
*Bluetooth*	IEEE802.15.1	24 Mbps (v3.0)	2.4 GHz	Low	10 m typical	Small networks Security, speed Easy access Flexibility	HAN	Challenge response scheme/CRC32
WiFi	EEE802.11x	11,54 to 300 Mbps outdoor	2.4 GHz 5 GHz	Very high	Up to100 m	Popular in HAN Speed, flexibility	HAN	4-Way handshake/ CRC32
*Z-Wave*	802.11	100Kbps	2.4GHz 868.42 MHz (EU)	Low	30 m indoor; 100 m outdoor	No interferences	HAN, NAN	AES128/ 32bit home I.D
*Zigbee*	IEEEE802.15.4	256 Kbps	2.4 GHz	Very low	10–100 m	Low cost Low consume Flexible topology	HAN,NAN	ENC-MIC-128 Encrypted key/ CRC16
*LPWAN*	SigFox LoRaWAN NB-IoT	0.3 to 50 kbit/s per channel	915 MHz	Low	10 km in rural settings	Low power Low cost	NAN,WAN	Symmetric key cryptography/AES 128b
6LoWPAN	IEEEE802.15.4	250 Kbps	2.4 GHz	Low	Up to 200 m	Low energy use	HAN, NAN	Symmetric key cryptography/AES 128b
*GSM/GPRS*	ETSI GSM EN 301349 EN 301347	14.4 Kbps (GSM) 114 Kbps (GPRS)	935 MHz Europe 1800 MHz	Low	Several Km	Low cost Signal quality	HAN, NAN WAN	64 bit A5/1 encryption/ Session key generation
*WLAN*	IEEE 802.11	150 Mbps	2.4 GHz Europe	Low	250m	Robustness	HAN, WAN	WEP, WPA, WPA2/ Open, Shared EAP
*5G*	5G Tech Tracker	Up to 20 Gbps	3400-3800 MHz awarding trial licenses (EU)	Very Low	46 m indoor; 92m outdoor	High speed Low latency	HAN, WAN	Symmetric key encryption/ Mobility management entity
*3G*	UMTS	Up to 14.4 Mbps	450,800 MHz 1.9 GHz	Low	Up to 100 m	Fast Data Transfer	HAN,WAN	CDMA2000/ Authentication and Key Agreement

**Table 6:** Hardware platforms.

Hardware	Features	Communication Transceivers	Operating System	Power Consumption	Strengths/Weakness
*Raspberry Pi 3*	1.2 GHz Quad Core BCM2837 64bit CPU 1GB	4 USB, Wi-Fi, Bluetooth, optional ZigBee and Z-Wave	Raspbian Ubuntu Windows 10	1.8 W	Open source platform; Use Python or C++; Cost: 50
*Arduino*	32 MHz Micro controller based on ATmega2560 32 kB	WiFi, Bluetooth, ZigBee, GSM	Processing-based	0.2W	Open source platform hardware/software; High flexibility. Cost: 30; Appliances compatibility
*BeagleBone*	720 MHz MR Cortex-A8 processor 512 MB	1 USB port, PLC, Bluetooth, Ethernet	Angstrom Linux	1 W	Open source platform similar to Raspberry; Easy setting up; Cost: 90
*RADXA*	ROCK Pi 4 is a Rockchip RK3399 based SBC six core ARM processor, 1GB	WiFi, Bluetooth 5.0, USB Port, GbE LAN	Linux	2.3 W	Open source platform; High flexibility; Cost:50
*Libelium* *Waspmote*	14.7 MHz ATmega1281 28 kB	1USB, 802.15.4/ZigBee LoRaWAN,WiFi PRO GSM/GPRS,4G modules	Linux	2 W	High flexibility; Starter kit:200; ZigBee,WiFi and LoRaWAN support
Xilinx Spartan FPGA	16 Mb SPI flash memory, 100 MHz	Ethernet, USB port	Linux	2 W	SH, Deep Learning, Autonomous System
PYNQ	Embedded systems Xilinx Zynq Systems on Chips (SoCs)	Bluetooth, Ethernet, USB port	Linux	2.3 W	IoT hardware development in Python
*Control4Home* *Automation*	Control4Home owners enjoy personalised smart living experiences	Bluetooth, WiFi Z-Wave and ZigBee	Licensed	-	Operation with internet connection; Not user installation
*Nexia*	Smart home automation system	Z-Wave	Licensed	-	No knowledge of installation required/ Only Z-Wave support; Low compatibility
*LG smart* *appliance*	Control key features on LG smart appliances from your smartphone	WiFi	Licensed	-	No knowledge of installation required/ Only for LG appliances; Closed source

## Abbreviations

The following abbreviations are used in this manuscript.

N	Consumer number
$A_{i}$	Appliance number
*i*	Consumer identifier
*j*	Appliance index
$a_{ij}$	Consumer *i*’s appliance identifier
*t*	Certain time
RW	24-hour supply vector from renewables
$t_{beg}$	Earliest start time appliance
$t_{end}$	Latest final time appliance
$t_{sched}$	Scheduled start time of appliance
D	Consumer demand
vD	Variable demand
fD	Fixed demand
CF	Consumer Flexibility
L	Duration of the planned operation of appliance $a_{ij}$ in the next day
SH	Smart Home
HEMS	Home Energy Manager System
HAN	Home Arena Network
NAN	Neighbour Area Network
WAN	Wide Area Network
IoT	Internet Of Things
ICT	Information and Communication Technologies
SG	Smart Grid
DSM	Demand System Manager
MILP	Mixed Integer Linear Programming
SA	Simulates Annealing
PSO	Particle Search Optimisation
GA	Genetics Algorithm
PS	Pattern Search
RR	Round-Robin
PLC	Power Line Carries
CC	Computational Cost
